# Exploring the mechanism of action of *Bidens pilosa* L. in combating hepatic fibrosis through network pharmacology and molecular docking: An observational study

**DOI:** 10.1097/MD.0000000000039725

**Published:** 2024-09-13

**Authors:** Jie Zhao, Mei Wang, Qing Yu, Sanhua Zhan, Mingyang Mao

**Affiliations:** a Department of Pharmacy, Tongling People’s Hospital, Tongling, Anhui Province, China; b Department of Pharmacy, Tongling Sixth People’s Hospital, Tongling, Anhui Province, China.

**Keywords:** *Bidens bipinnata* L, liver fibrosis, molecular docking, network pharmacology

## Abstract

Based on network pharmacology and molecular docking methods, to explore the possible targets and mechanisms of *Bidens pilosa* L. in treatment of liver fibrosis. The TCMSP, GeneCard, OMIM, TTD and DrugBank databases were used to obtain the targets of *Bidens pilosa L and* liver fibrosis, than the intersection targets were screened out by Venny 2.1.0, the protein-protein interaction (PPI) network and the core targets were obtained by the STRING database. Use Cytoscape3.7.2 software to draw the “traditional Chinese medicine-component-target-disease” network. The DAVID database platform was explored to analyze the biological process and pathway, and predict the anti-liver fibrosis mechanism of *Bidens pilosa* L. AutoDock and PyMol were used to verify the molecular docking between the active ingredients of *Bidens pilosa* L. and the core targets. Six active components of *Bidens pilosa* L. and 106 intersection targets were screened. PIK3R1, HSP90AA1, SRC, TP53, AKT1, RELA and other core targets were screened by PPI network analysis. The results of GO and KEGG enrichment analysis showed that the anti-liver fibrosis of *Bidens pilosa L* mainly involved in the regulation and negative regulation of apoptosis process, positive regulation of protein kinase B signal transduction, positive regulation of cell migration and other biological processes. Pathways acting on cancer, fluid shear stress and atherosclerosis, lipids and atherosclerosis, PI3K-AKT signaling pathway, MAPK signaling pathway and other signaling pathways. Molecular docking showed that the active components of *Bidens pilosa* L. displayed good binding activity with core target proteins, and the average binding energy was −7.47 kcal/mol. The possible mechanism of the active components against liver fibrosis is to regulate the PI3K-AKT, MAPK, and other signaling pathways by acting on core targets such as PIK3R1, HSP90AA1, SRC, TP53, AKT1, RELA, and induce the apoptosis of activated HSC cells to reverse and improve liver fibrosis.

## 1. Introduction

Hepatic fibrosis is characterized by the excessive deposition and abnormal distribution of the extracellular matrix in liver cells, induced by various pathogenic factors, leading to a pathological process that further progresses to disrupt liver structure and function.^[1]^ It is a critical step and important aspect in the progression of various chronic liver diseases to liver cirrhosis and cancer.^[2]^ Hence, early, proactive, and effective intervention is crucial in the treatment process, significantly impacting the delay or even reversal of its progression. However, due to the complex mechanisms of hepatic fibrosis, drugs targeting a single molecule or pathway are insufficiently effective against hepatic fibrosis, and currently, there are no definitive drugs available for its treatment. Traditional Chinese medicine, with its unique multi-component, multi-target, and multi-pathway synergistic regulation, has shown promising effects against hepatic fibrosis.^[3]^

*Bidens pilosa* L., an annual herbaceous plant of the Asteraceae family, is widely valued for its medicinal properties; the whole plant is usable, presenting a bitter taste, neutral properties, and non-toxicity. It is known for its heat-clearing, detoxifying, fatigue-dispersing, and swelling-reducing effects. Pharmacological studies showed that the whole plant of *Bidens pilosa* L. has significant efficacy and minimal side effects in treating hepatic fibrosis, hypertensive, diabetic activities.^[4,5]^ Research by Yuan Liping has also elucidated some of the anti-hepatic fibrosis targets and pathways of the active components in *Bidens pilosa* L.^[6]^ To further explore the active components, targets, and pathways of *Bidens pilosa* L. in combating hepatic fibrosis, this study employs network pharmacology and molecular docking to conduct an in-depth analysis, systematically elucidating the mechanism of action of *Bidens pilosa* L. in counteracting hepatic fibrosis and providing a reference for subsequent research.

## 2. Materials and methods

### 2.1. Selection of active components and targets of *Bidens pilosa* L

In the Traditional Chinese Medicine Systems Pharmacology Database (TCMSP, https://www.tcmsp-e.com/#/database), using “*Bidens pilosa* L.” as the search term, active components with an oral bioavailability (OB) ≥ 30% and drug-likeness (DL) ≥ 0.18 were selected. The targets of these active components were collected from the TCMSP and SwissTargetPrediction databases (http://www.swisstargetprediction.ch), deduplicated, and their protein names were converted to gene names using the UniProt database (https://www.uniprot.org) to obtain the final drug target genes.

### 2.2. Selection of liver fibrosis-related targets

Using “liver fibrosis” as a keyword, relevant genes were searched in the GeneCards, OMIM, TTD, and DrugBank databases, with GeneCards scores ≥ 10 as the filtering criterion. The collected data were deduplicated and standardized using the UniProt database to identify liver fibrosis-related target genes. The drug and disease target genes were imported into the Venn 2.1.0 platform to create a Venn diagram illustrating the intersection of drug and disease targets and to export the intersecting target data.

### 2.3. Construction of the PPI network

The intersecting target data were imported into the String database (https://cn.string-db.org/) for protein–protein interaction (PPI) analysis, setting the biological species to “Homo sapiens” and the confidence level to “highest confidence (0.9).” The protein–protein interaction network was exported and visualized using Cytoscape 3.7.2 software, ultimately producing a PPI network graph of the intersecting targets, which facilitated the identification of core targets.

### 2.4. Construction of the “*Bidens pilosa* L.-Active Component-Target-Liver Fibrosis” network

*Bidens pilosa*, its active components, intersecting targets, and liver fibrosis were imported into Cytoscape 3.7.2 software to construct the “*Bidens pilosa L*-Active Component-Target-Liver Fibrosis” network.

### 2.5. GO and KEGG pathway enrichment analysis

The DAVID database (https://david.ncifcrf.gov/tools.jsp) was utilized for GO functional and KEGG pathway enrichment analyses, applying *P* ≤ .05 and *q* ≤ 0.05 as filtering criteria. This yielded results for GO biological processes (BP) and KEGG signaling pathway enrichment, essential for understanding the main BP and signaling pathways through which *Bidens pilosa* exerts its anti-hepatic fibrosis effects.

### 2.6. Molecular docking

To elucidate the binding activity between the core targets and active components of *Bidens pilosa* L. in its anti-hepatic fibrosis action, the 2- and 3-dimensional structures of active components and core target proteins were downloaded from the PubChem and RCSB databases, respectively, saving them in PDB format. PyMOL was used to process the PDB molecules, and AutoDock Tools was employed for molecular docking of the active components and target proteins. This analysis determined the binding interactions between target protein receptors and small molecule ligands, selecting the most suitable conformations, with visualization conducted using PyMOL.^[7]^

## 3. Results

### 3.1. Selection and target prediction of active components of *Bidens pilosa* L

A search in the TCMSP database identified 62 active components of *Bidens pilosa* L. Using a selection criterion of OB ≥ 30% and DL ≥ 0.18, 6 active components were identified, as shown in Table 1. The TCMSP and SwissTargetPrediction databases were searched for corresponding targets of the active components. One component, MOL006441, did not match any targets. The data for the remaining active components’ targets were consolidated and deduplicated, with the UniProt database converting target protein names to gene names, resulting in 274 drug target genes.

**Table 1:** Active components of *Bidens pilosa* L.

MOL ID	Active components	OB (%)	DL
MOL006436	Okanin	98.81	0.2
MOL006442	(R)-2-(3,4-dihydroxyphenyl)-6,7-dihydroxybenzofuran-3 (2H)-one	57.1	0.21
MOL006441	Bidenphenol glucoside	55.9	0.61
MOL000098	quercetin	46.43	0.28
MOL006438	(2E)-2-(3,4-dihydroxybenzylidene)-6,7-dihydroxy-benzofuran-3-one	39.48	0.25
MOL000006	Luteolin	36.16	0.25

### 3.2. Prediction of liver fibrosis-related targets

**Figure 1.**
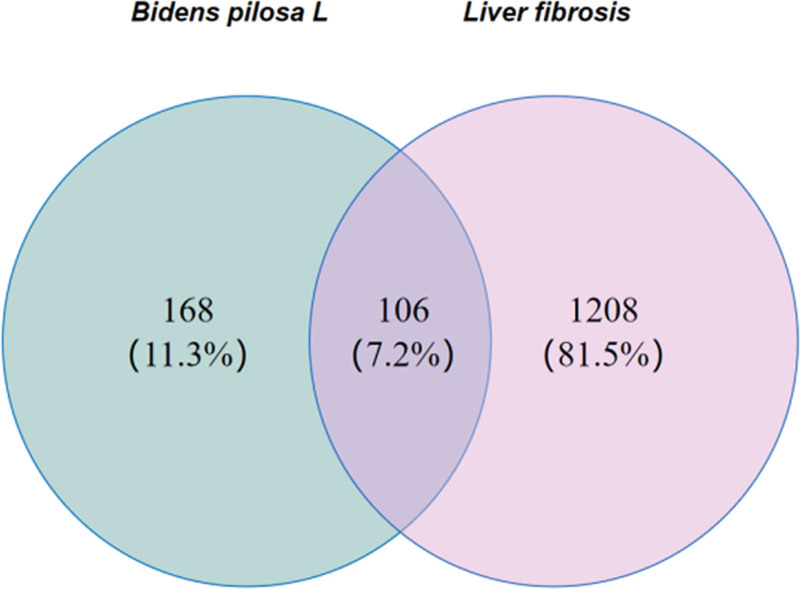
Venn diagram of drug and disease targets.

Searches in the GeneCards, OMIM, TTD, and DrugBank databases identified 1314 liver fibrosis-related target genes. Using Venny 2.1.0, an intersection of targets from the active components in *Bidens pilosa* L. and the disease-related targets yielded 106 intersecting targets, as illustrated in Figure 1.

### 3.3. Construction of the PPI network and selection of core targets

**Figure 2.**
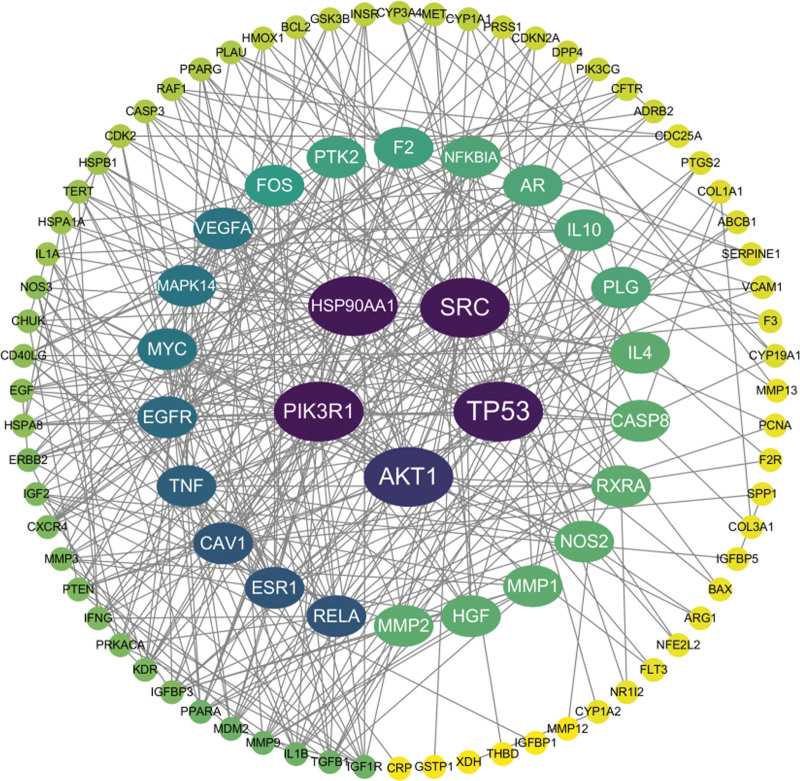
PPI network of drug–disease targets.

The intersecting target data were imported into the String database, and a PPI network of these targets was constructed using Cytoscape 3.7.2 software, as shown in Figure 2, comprising 106 nodes and forming 407 interaction relationships. PPI network analysis identified core targets such as PIK3R1, HSP90AA1, SRC, TP53, AKT1, and RELA in *Bidens pilosa* treatment of hepatic fibrosis.

### 3.4. “*Bidens pilosa* L.-Active Component-Target-Liver Fibrosis” Network

**Figure 3.**
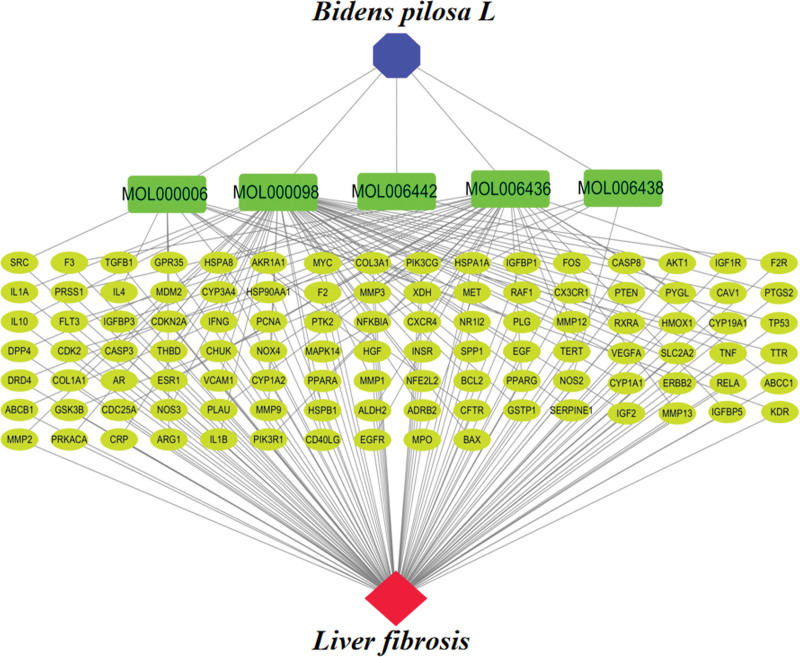
*Bidens pilosa L.*-active component-target-liver fibrosis network.

*Bidens pilosa*, its active components, intersecting targets, and liver fibrosis were integrated into Cytoscape 3.7.2 software to construct the “*Bidens pilosa* L.-Active Component-Target-Liver Fibrosis” network, as depicted in Figure 3.

### 3.5. Enrichment analysis

**Figure 4.**
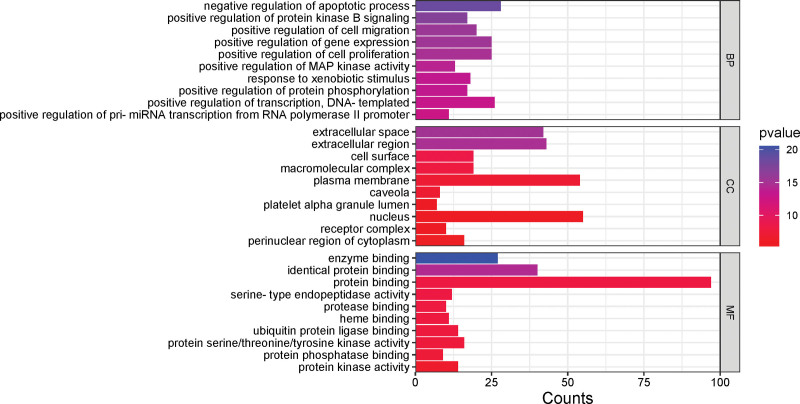
GO functional enrichment analysis.

GO functional enrichment analysis was conducted to investigate the BP, cellular components (CC), and molecular functions (MF) involved in the anti-hepatic fibrosis effects of *Bidens pilosa* active components. This analysis enriched 858 GO terms, including 69 CC mainly located in extracellular spaces, extracellular regions, cell surfaces, and macromolecular complexes; 668 BP, predominantly involved in the negative regulation of apoptosis, positive regulation of cell migration, positive regulation of protein kinase B signaling, and positive regulation of gene expression; and 121 MF, primarily related to enzyme binding, identical protein binding, protein binding, serine-type endopeptidase activity, and protease binding. The top 10 terms by *P*-value were selected for bar chart representation, as shown in Figure 4.

**Figure 5.**
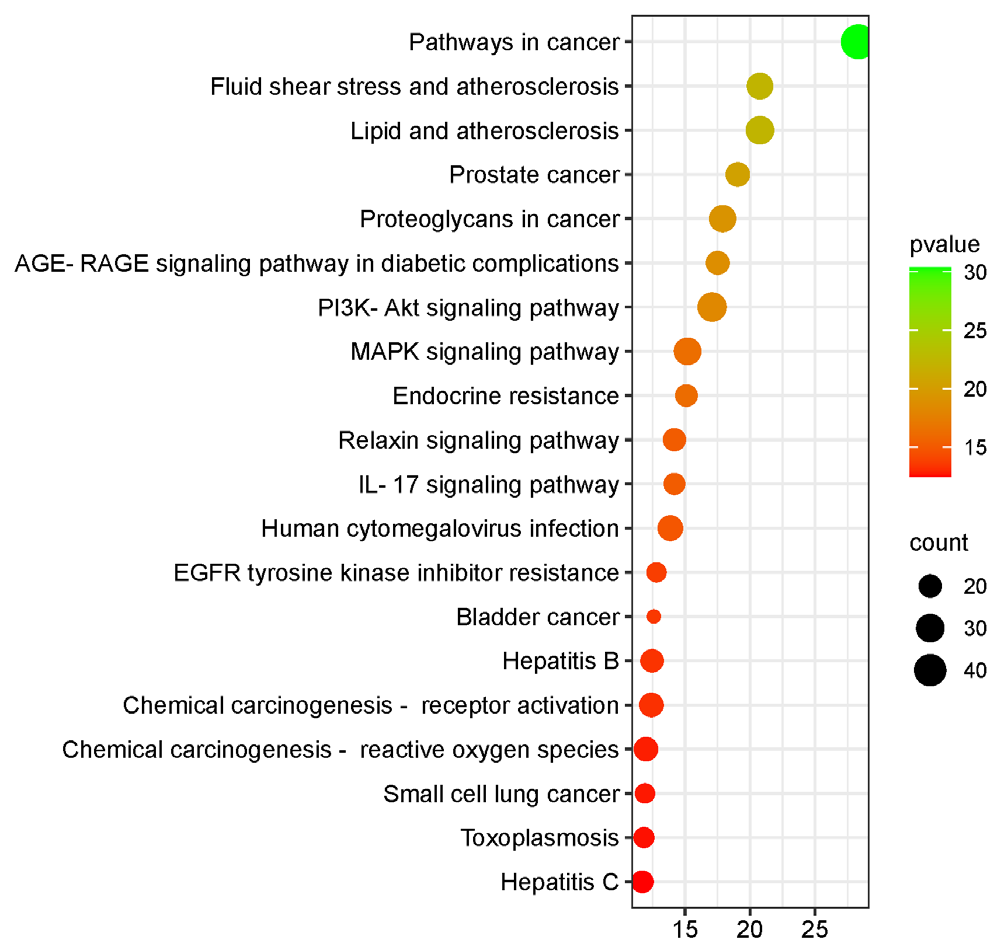
KEGG pathway enrichment analysis.

KEGG pathway enrichment analysis identified 164 signaling pathways. The top 20 enriched pathways by *P*-value were visualized in a bubble chart, where the color and size of the bubbles represent the significance of enrichment and the number of genes enriched in each pathway, respectively, as shown in Figure 5. Notably, *Bidens pilosa* L.’s anti-hepatic fibrosis effects are mainly associated with pathways related to cancer, fluid shear stress and atherosclerosis, lipid and atherosclerosis, PI3K-Akt, MAPK, prostate cancer, proteoglycans in cancer, and AGE-RAGE in diabetic complications.

### 3.6. Molecular docking

**Figure 6.**
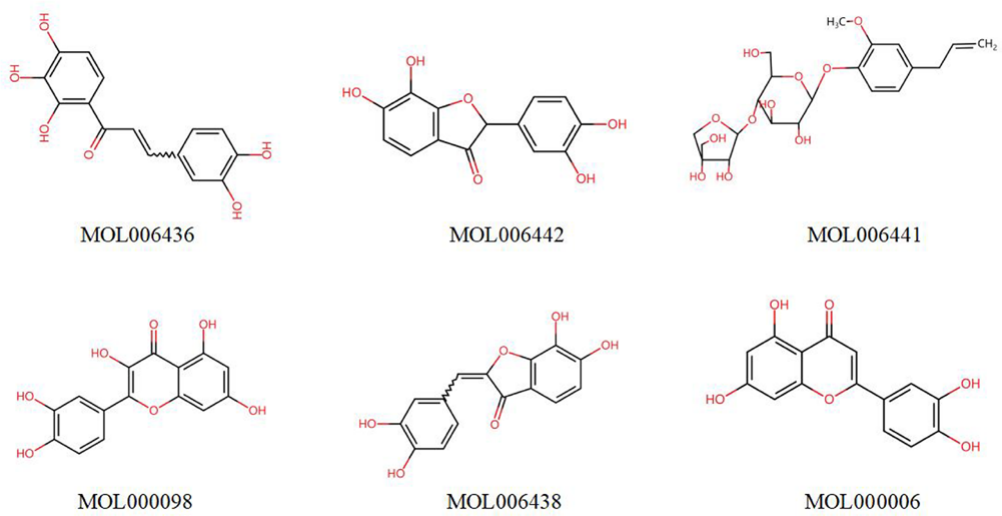
The 2-dimensional structures of the active components.

**Figure 7.**
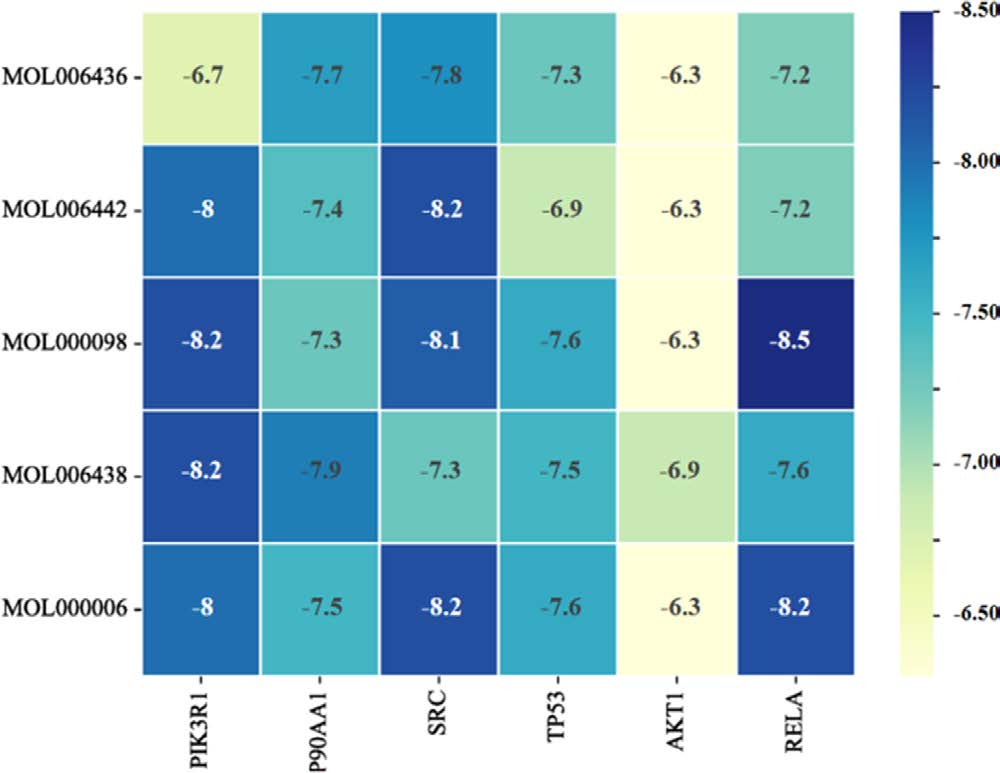
Molecular docking heat map.

**Figure 8.**
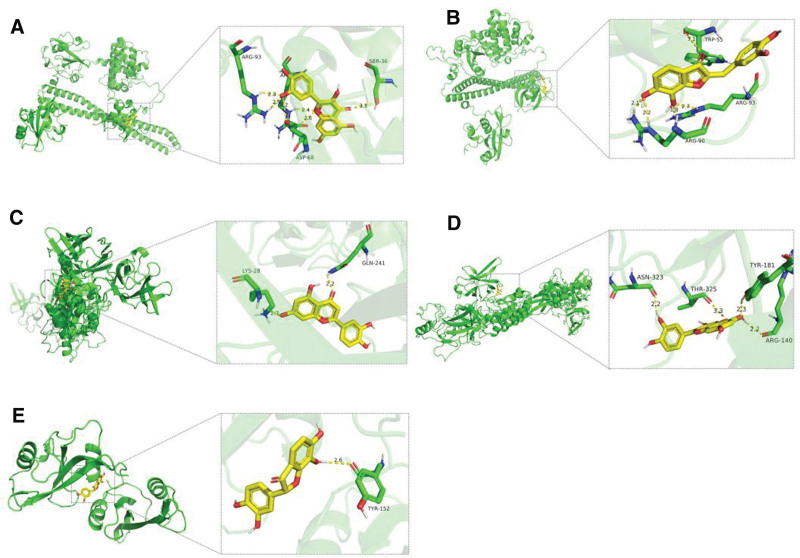
Molecular docking between the active ingredients of *Bidens pilosa L*. and the core target protein. (A) PIK3R1-MOL000098; (B) PIK3R1-MOL006438; (C) PIK3R1-MOL006438; (D) RELA-MOL000098; (E) SRC-MOL006442.

The selected core targets (PIK3R1, HSP90AA1, SRC, TP53, AKT1, RELA) were subjected to molecular docking with the active components of *Bidens pilosa* L. The 2-dimensional structures of these components were shown in Figure 6. The binding free energy was used to assess the affinity between receptors and ligands. A binding energy <0 indicates that the ligand and receptor protein can spontaneously bind, and a minimum binding energy less than −5.0 kcal/mol suggests a favorable docking between the ligand and receptor protein. The results showed an average binding energy of −7.47 kcal/mol, indicating good docking between all receptor proteins and small molecule ligands, as seen in Figure 7. Notably, the docking modes of PIK3R1-MOL000098, PIK3R1-MOL006438, RELA-MOL000006, RELA-MOL000098, and SRC-MOL006442 were particularly favorable, as shown in Figure 8.

## 4. Discussion

Hepatic fibrosis is the liver’s wound healing response to any chronic or acute liver injury,^[8]^ and the persistence of fibrosis can lead to end-stage liver disease, cirrhosis, and hepatocellular carcinoma.^[9–11]^ Studies indicate that 2% to 10% of patients with hepatitis B can develop cirrhosis, with the incidence of hepatic fibrosis rising globally.^[12]^ Currently, other than liver transplantation, there are no effective curative treatments for hepatic fibrosis, underscoring the urgent need to explore effective antifibrotic drugs. Recent advancements in antifibrotic drug research and treatment have been made in targeting various aspects of hepatic fibrosis, including inhibiting the activation and proliferation of hepatic stellate cells (HSCs), enhancing the activity of matrix metalloproteinases, inhibiting the activity of tissue inhibitors of metalloproteinases, suppressing inflammatory responses, and regulating immune responses, including herbal medicines. Leveraging the advantages of traditional Chinese medicine’s multi-target, multi-pathway regulatory mechanisms, this study employs network pharmacology and molecular docking to predict the targets and pathways of *Bidens pilosa* L. in combating hepatic fibrosis, providing direction and guidance for subsequent research.

This study primarily utilized various datasets to identify the active components of *Bidens pilosa* L. and their target points related to hepatic fibrosis. By intersecting these data, 106 common targets were identified for PPI network and “*Bidens pilosa L*-Active Component-Target-Liver Fibrosis” network analyses. The results revealed that the main active components of *Bidens pilosa* effective against hepatic fibrosis are quercetin and okanin, followed by luteolin, with core targets including PIK3R1, HSP90AA1, SRC, TP53, AKT1, and RELA. Quercetin, known for its anti-inflammatory, immune-regulating, antioxidant, and cardiovascular protective effects,^[13]^ has been found to activate the PI3K/Akt signaling pathway.^[14,15]^ The docking result of quercetin and RELA has the lowest binding energy of −8.2 kcal/mol, indicating that the combination was quite stable and played a crucial role in regulating PI3K/Akt pathway in the development of hepatic fibrosis. This pathway’s activation inhibits the proliferation and activation of HSCs, increasing apoptosis, thereby preventing and slowing the progression of hepatic fibrosis.^[16]^ Chong observed a notable reduction in AKT and p-AKT expression in hepatic fibrosis tissue compared to normal liver tissue, indicating PI3K/AKT pathway suppression.^[17]^ Since PI3K can phosphorylate its downstream target protein Akt, and p-AKT can regulate the synthesis of pro-apoptotic proteins caspase, Bad, and Bax, thereby inhibiting apoptosis, effectively activating the PI3K/AKT pathway could be crucial for protecting liver cells and reducing hepatic fibrosis. HSP90, involved in regulating the PI3K/AKT pathway, can ameliorate hepatic fibrosis when inhibited.^[15,18]^ Quercetin also inhibits NF-κB activation in a dose-dependent manner, reduces p38 and MAPK expression, and modulates Bcl-2/Bax signaling, slowing hepatic fibrosis progression.^[19]^ Meanwhile, RELA, a transcription factor related to the NF-κB family, plays a vital role in inhibiting inflammatory responses and regulating endothelial cell proliferation, apoptosis, and adhesion.^[20]^ In our study, we also found quercetin and RELA has the lowest binding energy of −8.5 kcal/mol, followed by luteolin −8.2 kcal/mol, indicating that the combination is the most stable. Yuan Liping found that the expression of NF-κB and TGF-β1 was upregulated in the liver tissue of rats with CCl4-induced hepatic fibrosis, while Bidens pilosa extract significantly reduced the expression of NF-κB and TGF-β1 in the liver tissue of these rats, enhancing the expression of P53 and bax proteins, thereby inducing apoptosis in HSCs.^[21]^ Since NF-κB is expressed only in activated HSCs, it is believed that Bidens pilosa extract can induce apoptosis in activated HSCs by inhibiting NF-κB expression, thus exerting an anti-hepatic fibrosis effect. Cummins using human and rat hepatic stellate cell lines, found that luteolin could inhibit HSC activation by downregulating the total amount of STAT3 and its phosphorylation levels, playing an anti-fibrotic role in the liver.^[22]^ Research on okanin anti-hepatic fibrosis effects is less common, but our results suggest that okanin may exert its anti-fibrotic effects by targeting PIK3R1, EGFR, MMP13, among others, necessitating further investigation. We further validated the binding affinity of *Bidens pilosa* L.’s active components with key targets PIK3R1, HSP90AA1, SRC, TP53, AKT1, RELA using molecular docking techniques, revealing that the minimum binding energies between the active components and key targets were all below −5.0 kcal/mol, with an average binding energy of −7.47 kcal/mol. This indicates effective docking between the ligand molecules of *Bidens pilosa* L.’s active components and receptor proteins, corroborating the rationality and accuracy of the network pharmacology analysis results.

## 5. Conclusion

In summary, the potential mechanism of *Bidens pilosa* L.’s active components in combating hepatic fibrosis involves acting on relevant targets and pathways, such as PIK3R1, HSP90AA1, SRC, TP53, AKT1, and RELA, which inhibit the proliferation and activation of HSCs and increase apoptosis, thereby reversing and improving hepatic fibrosis. This also verified the therapeutic effect of *Bidens pilosa* L. in clinical liver disease. In addition, our research will provide a reference for further research on the mechanism of *Bidens pilosa* L. in the treatment of hepatic fibrosis. However, this study is based solely on bioinformatics to systematically predict the active components, targets, and pathways of *Bidens pilosa* L.’s anti-hepatic fibrosis effects, necessitating further experimental investigation to explore the molecular mechanisms by which *Bidens pilosa* L. exerts its anti-fibrotic effects.

## Acknowledgments

This project was supported by Natural Science Key Project of Bengbu Medical College (No. 2023byzd039). Thanks to the cardiologist and nursing staff for their great support. Thanks to Professor Wang Mei for his guidance on this study.

## Author contributions

**Conceptualization:** Jie Zhao, Mei Wang.

**Data curation:** Jie Zhao.

**Formal analysis:** Jie Zhao.

**Investigation:** Sanhua Zhan, Mingyang Mao.

**Methodology:** Jie Zhao.

**Project administration:** Jie Zhao, Qing Yu.

**Software:** Jie Zhao, Qing Yu.

**Supervision:** Mei Wang.

**Validation:** Mei Wang.

**Visualization:** Mei Wang, Mingyang Mao.

**Writing – original draft:** Jie Zhao.

**Writing – review & editing:** Mei Wang.
